# Evaluating Antibacterial Efficacy and Biocompatibility of PAN Nanofibers Loaded with Diclofenac Sodium Salt

**DOI:** 10.3390/polym13040510

**Published:** 2021-02-08

**Authors:** Muhammad Nauman Sarwar, Azeem Ullah, Md. Kaiser Haider, Nadir Hussain, Sana Ullah, Motahira Hashmi, Muhammad Qamar Khan, Ick Soo Kim

**Affiliations:** 1 Nano Fusion Technology Research Group, Institute for Fiber Engineering (IFES), Interdisciplinary Cluster for Cutting Edge Research (ICCER), Shinshu University, Tokida 3-15-1, Ueda, Nagano 386-8567, Japan; nsoctober5@gmail.com (M.N.S.); 08tex101@gmail.com (A.U.); kaisershakil@yahoo.com (M.K.H.); engr.nadir712@hotmail.com (N.H.); sanamalik269@gmail.com (S.U.); motahirashah31@gmail.com (M.H.); 2 Nanotechnology Research Lab, Department of Textile & Clothing, Faculty of Engineering & Technology, National Textile University Karachi Campus, Karachi 74900, Pakistan; qamarkhan154@gmail.com

**Keywords:** electrospinning, biocompatibility, antibacterial, wound care, nanofibers

## Abstract

Side effects of the drugs’ oral administration led us to examine the possibility of using diclofenac sodium (DLF) in a polymeric drug delivery system based on electrospun polyacrylonitrile (PAN) nanofibers, which can be produced cost-effectively and with good applicability for transdermal treatments. The inclusion of DLF in PAN nanofibers increased the nanofiber diameter from ~200 nm to ~500 nm. This increase can be attributed to the increase in the spinning solution viscosity. FTIR spectra confirm the entrapment of the DLF into the PAN nanofibers. X-ray diffraction pattern showed that the inclusion of the DLF in the PAN nanofibers had caused the misalignment in the polymeric chains of the PAN, thus resulting in a decrease of the peak intensity at 2θ = 17^o^. The DLF loaded PAN nanofibers were efficient against the gram-positive *Staphylococcus aureus (S. aureus)* and gram-negative *Escherichia coli (E. coli)*, with maximum inhibition zone of 16 ± 0.46 mm for *E. coli* and 15.5 ± 0.28 mm for *S. aureus.* Good cell viability ~95% for L929 cells in more extended incubation periods was reported. A gradual release of DLF from the PAN nanofiber was observed and can be attributed to the stability of Pan in PBS medium. Cell adhesion micrographs show that cell-cell interaction is stronger than the cell-material interaction. This type of weak cell interaction with the wound dressing is particularly advantageous, as this will not disturb the wound surface during the nursing of the wound.

## 1. Introduction

Skin is the largest organ of our body. It is also the first defense line in protecting the body’s internal and most sensitive parts from the attack of bacteria, xenobiotics, and dehydration [1,2,3,4,5]. Any discontinuity in the structure of the skin may lead to a condition called a wound. A wound must be secured from external harms such as bacterial and fungal infections etc. Wound dressings serve as a barrier between the wound’s microenvironment and the external environment. For this purpose, wound dressings with antibacterial properties can provide an efficient barrier to pathogens.

Nano-encapsulation technique for drug delivery systems has yielded numerous benefits, such as easy handling, stability, protection against oxidation, control over the release profile, multi-drug delivery, reduced toxicity, and side effects of the drugs [6,7]. Nanofiber based drug delivery systems for the control and site specific drug release were developed by encapsulating drugs into polymeric delivery system in order to provide a controllable release of certain ratio of drugs at a proper targeted site over a fairly long duration of time [8].

The application of micro and nanofiber is gaining interest in biomedical field due to the acquired advantage of improved therapeutic index, possibility for localized delivery and reduced toxicity of drugs [9]. Nanotechnology can serve as a modest provision for avoiding the high systemic and topical drug dose as a preventive medicine source. At present, electrospinning is the predominant technique to develop wound dressing because of its simplicity, low cost, high production rate, and ability to create nanofibers having multiple morphological structures [10,11]. Besides, their porous structure, ability to absorb wound exudates, high specific surface area, and structure similarity to the natural extracellular matrix (ECM) nanofibers are widely used for tissue regeneration, wound dressing, and drug delivery system [12,13,14]. Electrospun nanofibers have found their applications in numerous fields such as wound healing [15], antibacterial [16,17], sensors [18], filtration [19], and other environmental application. Various delivery systems based on (natural & synthetic) polymers, liposomes, inorganic nanoparticles, etc., are generally used for wound dressing. Among these systems, the polymer-based system is widely practiced clinically [20,21]. A wide range of polymers (natural &synthetic) has been successfully electrospun for making wound dressing. Among various polymers, polyacrylonitrile (PAN) is widely used for wound dressing because; it possesses outstanding features such as light resistance, chemical stability, non-toxicity, mechanical elasticity, and electrospinabilty [22,23]. PAN or its composite with other polymers combined with different drugs or nanoparticles have been used for medical applications. Many researchers have successfully synthesized different nanofiber composite and drug-loaded delivery systems by incorporating other materials such as acyclovir, TiO_2_, AgCl, and silica [24,25,26].

Diclofenac (DLF) is a non-steroidal anti-inflammatory drug (NSAID), which is a benzene-acetic acid derivative and considered one of the best NSAID’s medicine for the treatment of acute and chronic Painful and inflammatory injuries [27,28]. Nano packaging of the drugs can enhance their medicinal efficacy, specificity, tolerability, therapeutic index, and polymer-based nano-carriers can further protect the drugs from degradation and reduce the toxicity or side effects. Though NSAID DLF has potent anti-inflammatory, analgesic, and antipyretic properties, but also is susceptible to risks of toxicity and other adverse effects. Encapsulating DLF in Eudragit^®^ L100-55 has been reported to reduce these drawbacks [29]. Huh, et al. proposed a surgical suture loaded with DLS for sustainable release as local pain relief of the surgical wound. DLF was loaded into the biodegradable poly lactic-co-glycolic acid stand and then braided with a surgical suture VICRYL. In this way, a sustained release for 10 days of DLF was recorded while maintaining the wound closure’s mechanical strength [30]. In another work, DLF loaded zein nanoparticles were prepared by the anti-solvent precipitation method and loaded into PVA nanofibers. A more controlled release of DLF was achieved by embedding the zein nanoparticles in PVA nanofibers. The results suggested that embedding DLF in a polymeric system improves its cell attachment and proliferation [31]. Nanonets of DLF loaded polycaprolactone and chitosan were prepared by electrospinning. Because of the nanofiber net’s slow degradation, the release was mostly governed by diffusion or permeation. The cell viability and imaging data indicate that NIH 3T3 cells experienced no toxicity in the DLF- nanonets structures [32]. Most of these studies carried in the near past are based on the release and toxicity study of the DLF loaded polymeric systems.

The side effects of oral administration of drugs have led us to investigate the possibility of encapsulating DLF in a polymeric drug delivery system based on PAN nanofibers which can be produced cheaply and having high applicability to be employed for transdermal delivery of drugs on the wound site. Besides, the polymer selection was based upon the high drug loading capacity, good mechanical strength, and prolong release characteristics [33]. The present work describes the preparation of antibacterial DLF loaded nanofibers using PAN as the filament-forming matrix, aimed for transdermal delivery. The prepared nanofibers were subjected to physicochemical, scanning electron microscopy, X-ray diffraction, in vitro release, antibacterial efficacy, and in-vitro cell proliferation analysis.

## 2. Materials and Methods

### 2.1. Materials

Polyacrylonitrile (PAN) (MW 150,000) procured from Sigma Aldrich (St. Louis, MO, USA). Diclofenac sodium salt (DLF) was purchased from Sigma Aldrich (St. Louis, MO, USA). N, N-Dimethylformamide (DMF) (99.8%) was obtained from Fujifilm Wako Chemicals Ltd. (Osaka, Japan). All chemicals were used as received without any further purification.

### 2.2. Preparation of PAN/Diclofenac Electrospinning Solution

The 10% *w*/*w* solution of polyacrylonitrile (PAN) was prepared in N, N-Dimethylformamide (DMF) with continuous stirring. After stirring the PAN solution for 5 h at ambient temperature, Diclofenac sodium salt (DLF) was added to the polymer solution with 3, 4, 5, and 6% by weight of the PAN. Afterward, the stirring was continued for 2 h to prepare the final PAN/ DLF solutions for electrospinning. A brief overview of the electrospinning solutions is given in Table 1.

### 2.3. Electrospinning Process

A horizontal electrospinning module was set up by using a high voltage power supply (Har-100*12, Matsusada Co., Tokyo, Japan) and a syringe pump (KDS-100, KD Scientific, Holliston, USA). The electrospinning solutions were fed in a 25 mL syringe equipped with a 22 gauge needle. A high voltage of 12.5 kV was applied across the needle and collector covered with butter paper. A fed rate adjusted at 0.7 mL/h, and a collector to needle distance was 130 mm. All samples were spun for 20 h to ensure a uniform thickness of the fibrous mats.

### 2.4. Surface Morphology Characterizations

The surface morphology of Nanofibers was evaluated by using scanning electron microscopy (SEM, JSM-5300, JEOL Ltd., Nagano, Japan) with an accelerating voltage of 12 kV and TEM (JEM-2100 JEOL, Nagano, Japan) accelerated with 200 kV. The average diameter distribution of nanofibers was calculated by image analysis software (ImageJ, version 1.49). The nanofiber specimen were sputter coated with platinum before subjecting to the SEM imagery.

### 2.5. Physicochemical Characterizations

An attenuated total reflectance Fourier transform infrared (ATR-FTIR) spectroscopy (Prestige-21, Shimadzu, Japan) was used to determine the nanofiber dressing chemical composition. FTIR spectra were scanned over the wavelength range 4000–600 cm^−1^ at room temperature, and a spectrum was accumulated over 20 scans.

### 2.6. XRD Characterizations

The X-ray diffractograms (XRD) of the neat and DLF loaded fibrous mats were determined by an X-ray diffractometer (Miniflex 300, Rigaku Co., Ltd., Tokyo, Japan) operating at 30 kV and 500 mA. Diffractions were detected in the 2θ = 5°–80° range with a step of 0.05°.

### 2.7. The In-Vitro Release Behavior of DLF from the PAN Nanofibrous Mats

The in vitro release of DLF from the DLF/PAN nanofibrous mats was analyzed by the total immersion method in phosphate buffer (pH 7.4) containing 0.5% Tween 80 and 3% methanol (PTM buffer solution). The release medium was kept at 37 °C to mimic the body core temperature. Each specimen was immersed in 30 mL of releasing medium, and the dissolution time was 72 h. At a specific time interval, 1 mL of the release media was extracted, and the bath was replenished with the fresh buffer solution to keep the bath conditions constant. The amount of DLF in the media was determined by UV-spectroscopy at the characteristic wavelength of 276 nm (λmax). The absorbance spectra of the DLF at certain time intervals were converted into concentration using the calibration curve; y = 0.5984x^2^ + 11.04x − 4.789.

### 2.8. Antibacterial Efficacy

The antibacterial characteristics of fabricated electrospun nanofibers dressing were assessed against gram-positive *Staphylococcus aureus (S. aureus)* and gram-negative *Escherichia coli (E. coli)* bacteria by the qualitative method. *S. aureus* (ATCC 29213) and *E. coli* (ATCC 25922) strains were provided by Gene Research Centre, Shinshu University. A 100 μL of 10^−5^ dilution of overnight cultured *E. coli* and *S. aureus* were spread over the agar plates. The fiber mats were UV sterilized for 6 h before subjecting to an antibacterial test. The fiber mat samples (10 mm, diameter) were gently placed over the agar plates and incubated overnight. The agar plates were examined for bacterial growth interruption underneath and along the border for the inhibition zone.

### 2.9. Cell Viability and Adhesion

The L929 mouse normal fibroblast cell line mitochondrial activity was measured by WST-1 colorimetric assay using ISO 10993-5 standard. L929 cell line was provide by Gene research Centre, Shinshu University. In toxicity studies, the use of continuous cell lines, such as L929 mouse fibroblast is favored because of their biological response, ease of production, and easy control of their culture [34]. L929 cells were cultured in Dulbecco modified eagle medium (DMEM) accompanying 10% fetal bovine serum (FBS) in a humidified incubator at 37 °C and 5% CO_2_ environment. The fiber mats were sterilized in 70% ethanol for 30 min and then washed with PBS to remove any impurity. The sterilized mats were placed in 96 well glass culture plate. The culture plate was seeded with a density of 1 × 10^3^ cells per well. At the predetermined times, 10 µL WST −1 was added to each well and incubated for two hours. Absorbance was measured at 440 nm using a microplate reader (Thermos Scientific, Multiskan FC instruments) to indicate proliferation. Readings were measured in triplicate, and averages were recorded.

The cell-cell and cell-material interactions were observed with SEM micrographs. L929 cells were cultured with nanofiber mats for 72 h and were fixed on the mats with 4% paraformaldehyde at 4 °C. The mats were dehydrated using ethanol and dried in a vacuum oven. The nanofiber specimen were sputter coated with platinum before subjecting to the SEM imagery. The SEM micrographs were taken at a resolution of 300×.

### 2.10. Statistical Analysis

All statistical analysis regression equations and correlation coefficients were carried out using MINITAB 17 ^®^ statistical software [35].

## 3. Results and Discussion

### 3.1. Morphology and Diameter Analysis

**Figure 1:**
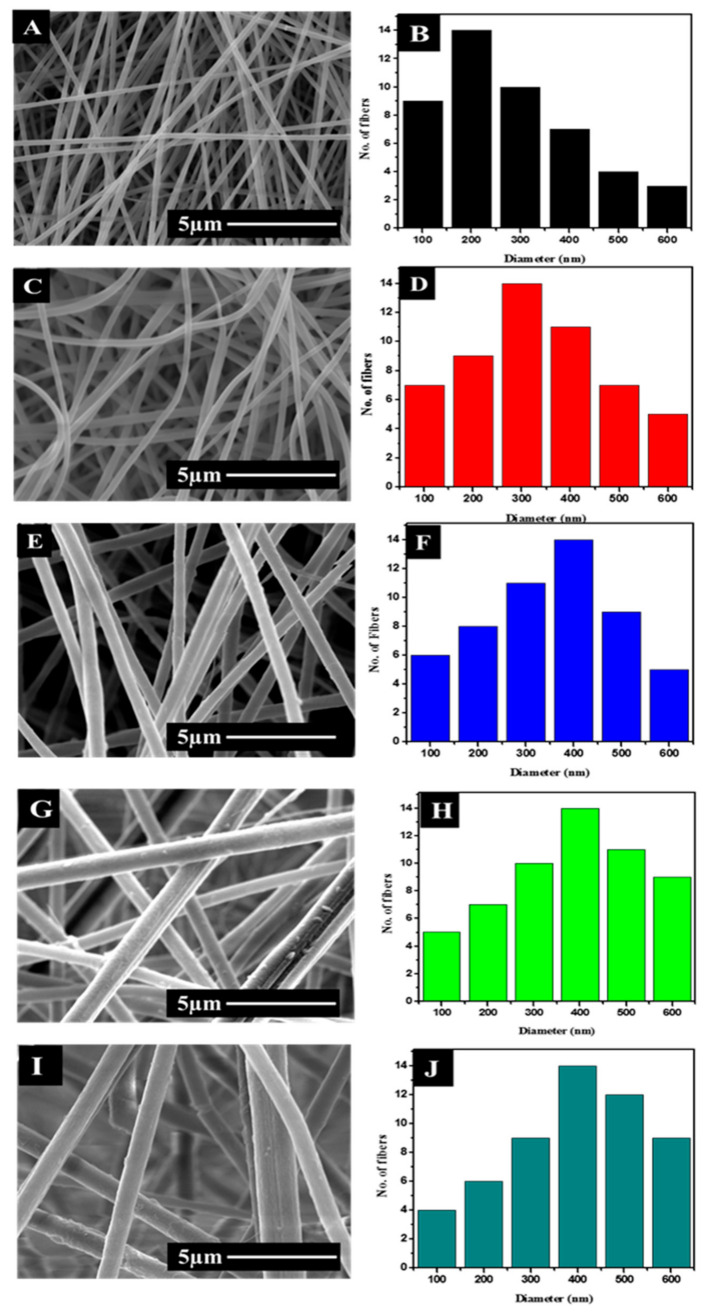
SEM micrographs and diameter histograms of S1 (**A**,**B**), S2 (**C**,**D**), S3 (**E**,**F**), S4 (**G**,**H**), and S5 (**I**,**J**).

SEM micrographs (Figure 1) were evaluated to analyze any difference in the surface morphology and diameter of the DLF loaded PAN nanofibers compared to pristine PAN nanofibers. The SEM micrographs confirm that uniform, bead-free continuous nanofibers were obtained in all cases. The pure PAN fibers showed an average diameter of ~215 ± 50 nm. The inclusion of DLF in the PAN nanofibers increased the nanofiber diameter. The increase in fiber diameter was in proportion to the amount of the DLF loaded into the fibers. The DLF loaded fibers showed an average diameter of ~279 ± 60, ~432 ± 30, ~467 ± 40, and ~489 ± 50 nm for S2, S3, S4, and S5. Though DLF salt can increase the electrical conductivity of the PAN solution and thereby should enhance the electrical drawing of the spinning jet. But with the addition of the DLF to the PAN solution the viscosity of the solution increased, and this increase in the viscosity counteracted the influence of the electrical conductivity and gradually had a greater influence on the increase in fiber diameter. The increase in nanofiber diameter by the inclusion of DLF is in good agreement with previously reported work [29].

### 3.2. Study of XRD Spectra

**Figure 2:**
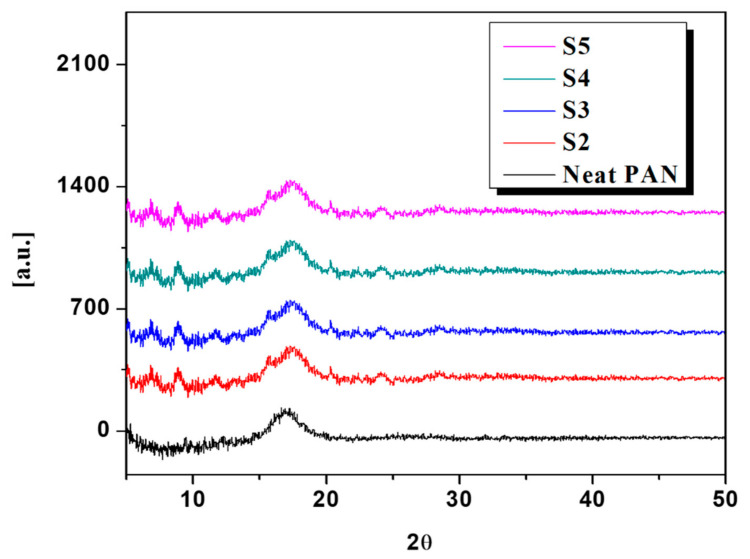
XRD diffractograms of neat (S1) and DLF loaded PAN nanofibers (S2, S3, S4, and S5).

Figure 2 represents the XRD diffractograms of the pristine and DLF loaded PAN nanofibers. The XRD pattern of the virgin PAN fibers showed a characteristic peak at 2θ = 17°. The peak at 2θ = 17° was associated with the hexagonal crystal lattice of PAN. The extraneous substances in a pure material result in a change in its molecular packing and is a fact [36]. DLF loaded PAN nanofibers, a characteristic peak at a shorter angle 2θ = 7.1° appeared, associated with the DLF and confirmed by the literature [37]. In addition to the appearance of a new peak at 2θ = 7.1°, the intensity of the PAN peak at 2θ = 17° in DLF loaded fibers also decreases. The addition of DLF could have caused a change in the molecular packing of the PAN, thus resulting in misalignment of the polymer microstructure.

### 3.3. Physicochemical Analysis

**Figure 3:**
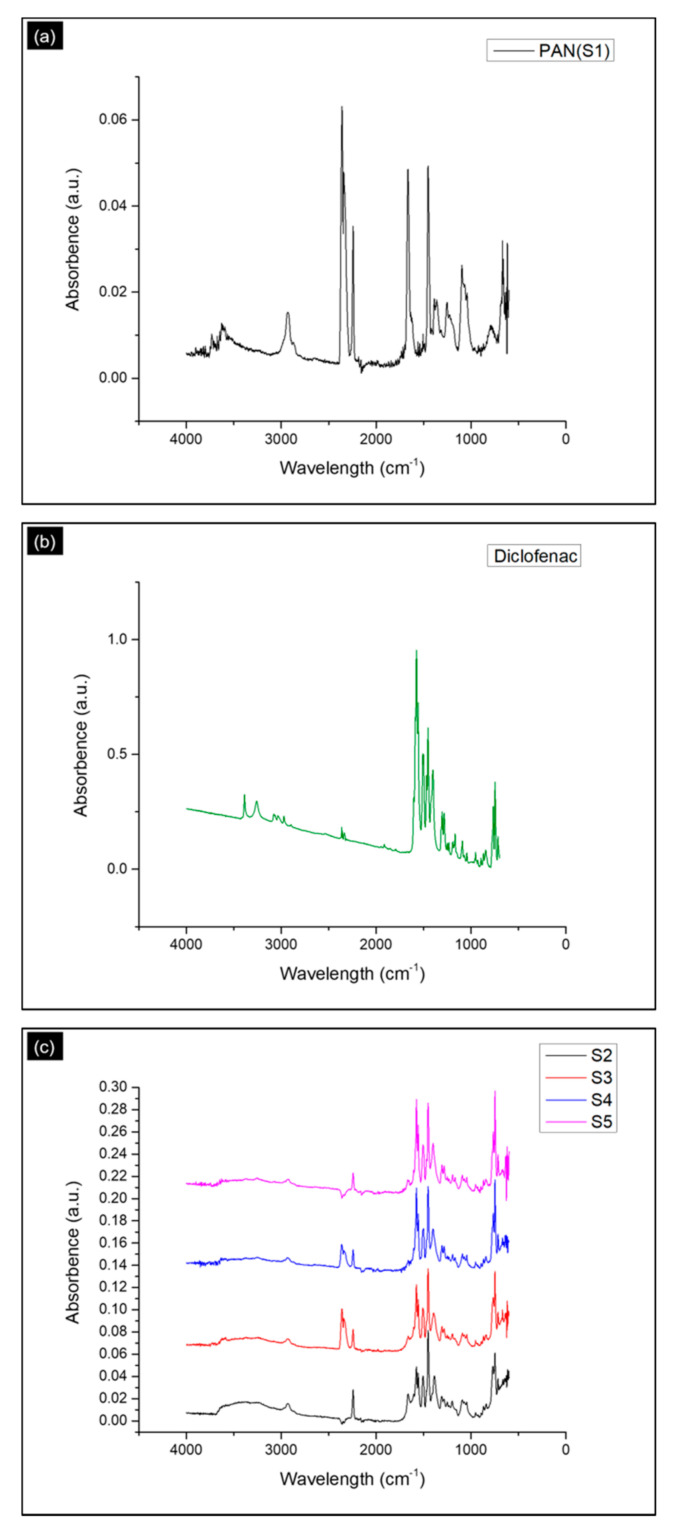
ATR-FTIR spectrum of (**a**) virgin PAN (S1), (**b**) Diclofenac sodium salt and (**c**) DLF loaded PAN nanofibers (S2, S3, S4, and S5).

Figure 3 represents the ATR-FTIR spectrum of the pure PAN, DLF, and DLF loaded PAN nanofibers. In Figure 3a, we can observe the sharp peaks at 2931, 2276, and 1451 cm^−1^. These peaks are due to the stretching frequencies of methylene (-CH2), nitrile (-CN), and bending vibrations of the methylene groups, respectively present in the PAN polymer backbone [38,39].

In DLF salt -NH and -NH-O stretching are visible at wavenumber 3407 and 3276 cm^−1^. The DLF also exhibited the two identifying bands of carboxylate stretching vibration at 1567 cm^−1^ (asymmetric) and at 1401 cm^−1^ (symmetric). Phenyl rings stretching vibrations are intense in the range of 1600–1450 cm^−1^ [40].

The spectra area between 1700–1000 cm^−1^ is quite similar to the ranges of pure materials. The only difference observed in the DLF loaded PAN nanofibers’ spectra from the Pure PAN nanofibers is in the region of 3500–3000 cm^−1^. The appearance of these peaks could be due to -NH and NH-O constituents of the DLF.

### 3.4. Antibacterial Activity

**Figure 4:**
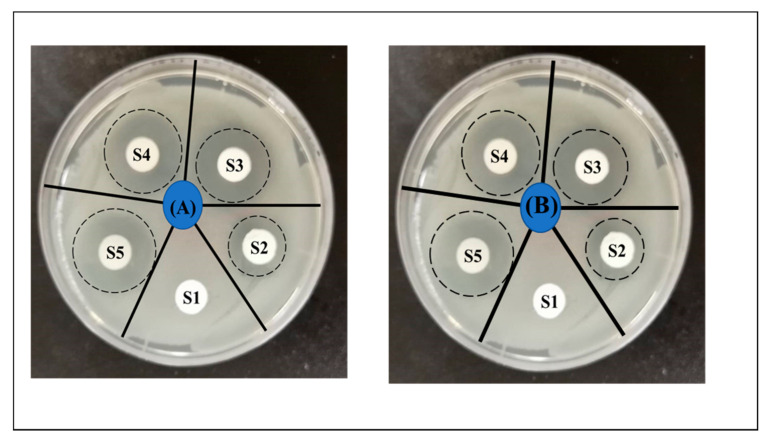
Bactericidal efficacy of DLF loaded PAN nanofiber (S2, S3, S4, and S5) using agar disc diffusion test (**A**) *E. coli* and (**B**) *S. aureus*.

The antibacterial efficiency of the pristine and DLF loaded PAN nanofiber was analyzed against the gram-negative (*E. coli*)(A) and gram-positive (*S. aureus*)(B). The chosen bacterial strains are the most frequent infection-causing bacteria to the soft tissues and results in the delayed wound healing. Most of the clinically acquired infections are resultant to *Staphylococcus aureus*. While *Escherichia coli* is considered as a primary source for infection in the burn wounds [41]. The exact mechanism of the antibacterial activity of diclofenac is unclear. However studies have proposed that the NSAID inhibits the bacterial DNA synthesis [42] or impairment of the membrane activity [43]. Figure 4 shows the halo created around the DLF loaded PAN nanofiber discs. These halos are the indication of the bacterial growth interruption. The halos were measured using ImageJ software and are recorded in millimeters and are given in Table 2. As the amount of DLF in the PAN nanofiber increases, the halo diameter increases. The largest inhibition zone was recorded for S5 with 16 ± 0.46 mm and 15.5 ± 0.28 mm for *E. coli* and *S. aureus*.

### 3.5. Cell Viability and Cell Adhesion

**Figure 5:**
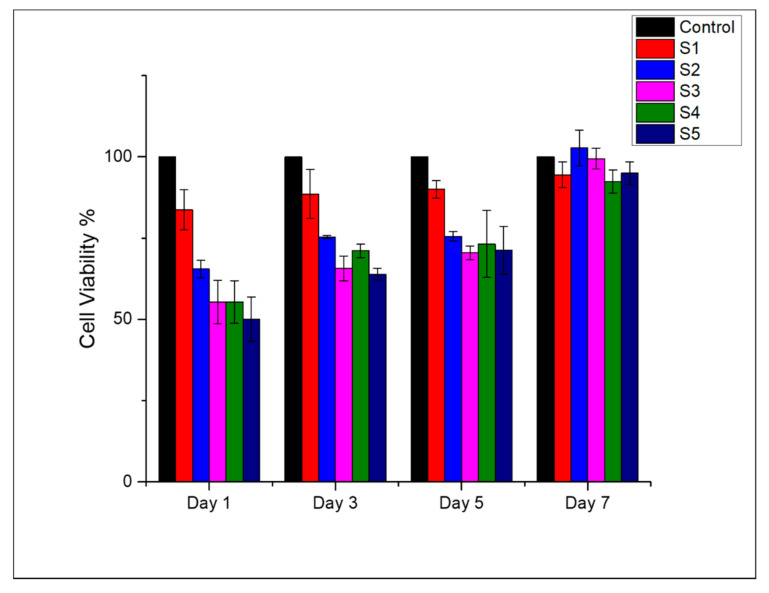
Cell viability of L929 cell line by mitochondrial activity using WST-1 assay, cultured for 1, 3, 5, and 7 days in presence of neat (S1) and DLF loaded PAN nanofibers (S2, S3, S4, and S5).

It is essential that the wound dressing should be biocompatible. Virgin and DLF loaded PAN nanofibers were subjected to the colorimetric WST-1 assay to determine the mitochondrial activity of the L929 cells. The analysis was carried out according to the standard ISO 10993-5 method. Figure 5 shows the viability of the L929 cell in the presence of DLF loaded and pure PAN nanofibers at 1, 3, 5, and 7 days of incubation. For the shorter period of incubation, the samples showed moderate toxicity ranging between ~50–70%. The DLF loaded mats showed a higher cell viability for more extended incubation periods ranging between ~75–100%, indicating good biocompatibility of the composite dressing. Furthermore, it can be said that the addition of DLF did not adversely affect the biocompatibility of the PAN nanofibers.

**Figure 6:**
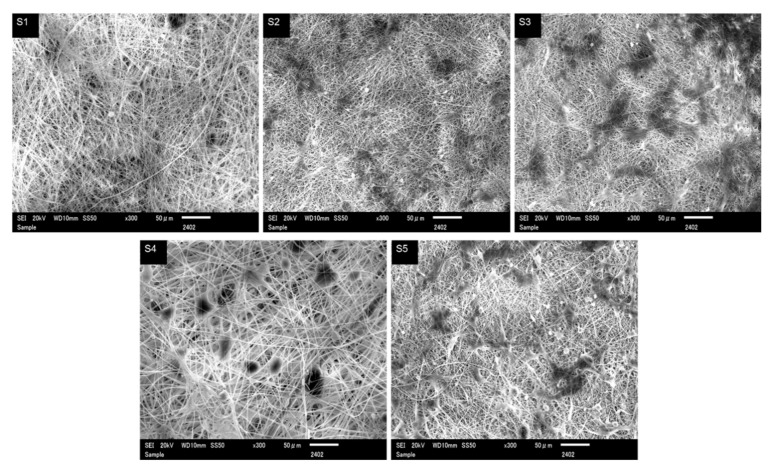
Cell attachment of L929 cells cultured for 7days in presence of neat (**S1**) and DLF loaded PAN nanofibers (**S2**– **S5**).

To determine the morphological differences, cell-cell and cell-material interactions were investigated by SEM imagery. The represented micrographs are shown in Figure 6. Interestingly cells were found in cluster form over the pure and DLF loaded PAN nanofiber mats. From the results, it is clear that the cell-cell interaction is stronger than the cell-material interaction, which results in a week attachment of L929 cell with the nanofibers, and cells are seen more in an agglomerated form over the dressing surface. This type of weak cell interaction with the wound dressing is particularly advantageous, as this will not disturb the wound surface during the nursing of the wound. This week cell-material interaction could also be a result of the intrinsic hydrophobic nature of the PAN nanofibers.

### 3.6. The Release Profile of DLF in PTM

**Figure 7:**
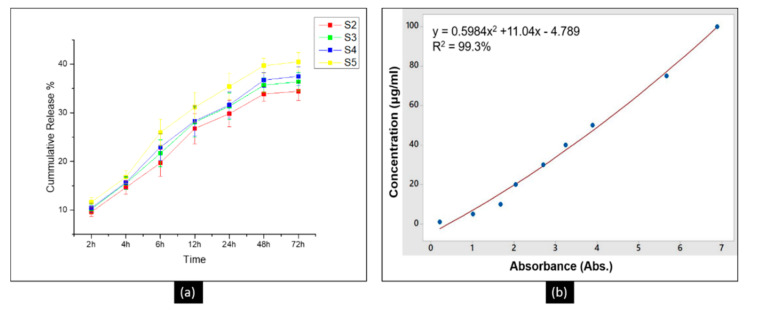
(**a**) In vitro release profile of DLF from PAN nanofibers (S2, S3, S4, and S5) in PBS, (**b**) calibration curve of the DLF in PBS showing regression model and coefficient of determination (R^2^).

Drug release profile of the DLF from the PAN nanofibers was investigated by immersing PAN/DLF nanofibers in PTM buffer solution, pH (7.4), at 37 °C. The drug release profile are demonstrated in Figure 7. It can be observed that DLF gradually released from the PAN nanofibers. At first the release rate was fast. This quick release is due to the diffusion of DLF from the surface of the PAN nanofibers. The gradual release of DLF continued until 48 h. After 48 h time interval the release almost become constant. It was observed that initial rapid release was dependent upon the amount of DLF loaded into the PAN nanofibers. The gradual release of DLF from PAN nanofibers is attributed to the chemical stability of PAN in the PBS release media. Based on the drug release profile we can say that the primary release of DLF from the PAN nanofiber is governed by diffusion and permeation from the surface of the nanofibers. This slow release is beneficial as PAN nanofiber will retain the bioactive agent throughout their useful life. Similar results were obtained with other drugs loaded into PAN nanofibers [33].

### 3.7. Practical Implications and Future Perspective

The produced nanofibers were aimed for providing efficient antibacterial activity against both gram positive and gram negative bacterial strains, along with the added advantage of the anti-inflammatory and analgesic properties of the DLF. The release of the PAN/DLF nanofiber system was suitable for the acute open wounds. Where this nanofiber dressing can provide efficient barrier properties. Moreover, the drug release was gradual and can be site specific over a fairly long duration of time.

The solubility of the drug in the polymeric system is of utmost importance and determines the bioavailability and drug distribution through the delivery system. Drug solubility has a major influence over the possible permeation across the biological membranes. The usage of the NSAID drugs as an effective antibacterial agent is relatively new and very few studies have been conducted for their effective encapsulation and targeted site delivery. So, different polymeric systems can be examined and in vitro alongside in vivo studies should be conducted to further validate the synergistic effect of NSAIDs as effective antibacterial.

## 4. Conclusions

Uniform bead-free continuous DLF loaded PAN nanofibers were successfully produced on horizontal electrospinning set up at a high voltage of 12.5 kV. FTIR spectrum and the appearance of a new peak at 7.1o in XRD diffractogram indicates the entrapment of the DLF in the PAN nanofiber matrices after electrospinning. The PAN nanofiber diameters increased proportionally to the increase in the amount of the DLF. The inclusion of DLF results in the polymer chain misalignment in the PAN and can be seen with decreased XRD peak intensity from the XRD diffractogram. DLF loaded nanofibers were efficient in inhibiting the bacterial growth of both *E. coli* and *S. aureus*. The inclusion of DLF in the PAN does not hinder the L929 cell proliferation. The SEM micrographs showed that the cell-cell interaction is stronger than the cell-material interaction, which is vital during wound nursing. DLF loaded PAN nanofiber showed a slow release with a maximum of ~40% release at 72 h time interval.

Based on the results it can be inferred that the produced nanofibers can provide an adequate antibacterial barrier for the acute wounds, along with a sustained release for long duration. Furthermore, the in vitro test revealed that the cell-cell interaction is stronger than the cell-material interaction, specifically owing to the inherent hydrophobic nature of the PAN nanofibers. The future prospect holds to promulgate the efficacy of the PAN/DLF nanofibers by undergoing extensive in vivo analysis.

## Figures and Tables

**Table 1:** Composition of electrospinning solution.

Sample No.	Weight Ratio (%)	
	PAN	DLF
**S1**	100	0
**S2**	97	3
**S3**	96	4
**S4**	95	5
**S5**	94	6

**Table 2:** Bacterial growth inhibition (zone of inhibition) for DLF loaded PAN nanofibers.

Samples Labels	Zone of Inhibition (mm)	
	*E. coli*	*S. aureus*
**S1(neat PAN)**	0	0
**S2(3% DLF)**	7 ± 0.15	8 ± 0.17
**S3(4% DLF)**	10.5 ± 0.19	12 ± 0.27
**S4(5% DLF)**	14 ± 0.38	15 ± 0.59
**S5(6% DLF)**	16 ± 0.46	15.5 ± 0.28

## Data Availability

The data can be requested from the corresponding author of the article.
