# Correction: Sarwar et al. Evaluating Antibacterial Efficacy and Biocompatibility of PAN Nanofibers Loaded with Diclofenac Sodium Salt. *Polymers* 2021, *13*, 510

**DOI:** 10.3390/polym17162170

**Published:** 2025-08-08

**Authors:** Muhammad Nauman Sarwar, Azeem Ullah, Md. Kaiser Haider, Nadir Hussain, Sana Ullah, Motahira Hashmi, Muhammad Qamar Khan, Ick Soo Kim

**Affiliations:** 1 Nano Fusion Technology Research Group, Institute for Fiber Engineering (IFES), Interdisciplinary Cluster for Cutting Edge Research (ICCER), Shinshu University, Tokida 3-15-1, Ueda, Nagano 386-8567, Japan; nsoctober5@gmail.com (M.N.S.); 08tex101@gmail.com (A.U.); kaisershakil@yahoo.com (M.K.H.); engr.nadir712@hotmail.com (N.H.); sanamalik269@gmail.com (S.U.); motahirashah31@gmail.com (M.H.); 2 Nanotechnology Research Lab, Department of Textile & Clothing, Faculty of Engineering & Technology, National Textile University Karachi Campus, Karachi 74900, Pakistan; qamarkhan154@gmail.com

## Error in Figure

In the originally published manuscript [1], an error was made in Figure 2, where the data was erroneously plotted using the same columns.

The corrected version of Figure 2 is provided below. The authors confirm that this error does not affect the scientific conclusions of the study.

This correction has been reviewed and approved by the Academic Editor. The original publication has also been updated accordingly.

We apologize for any confusion this may have caused.

## Figures and Tables

**Figure 2:**
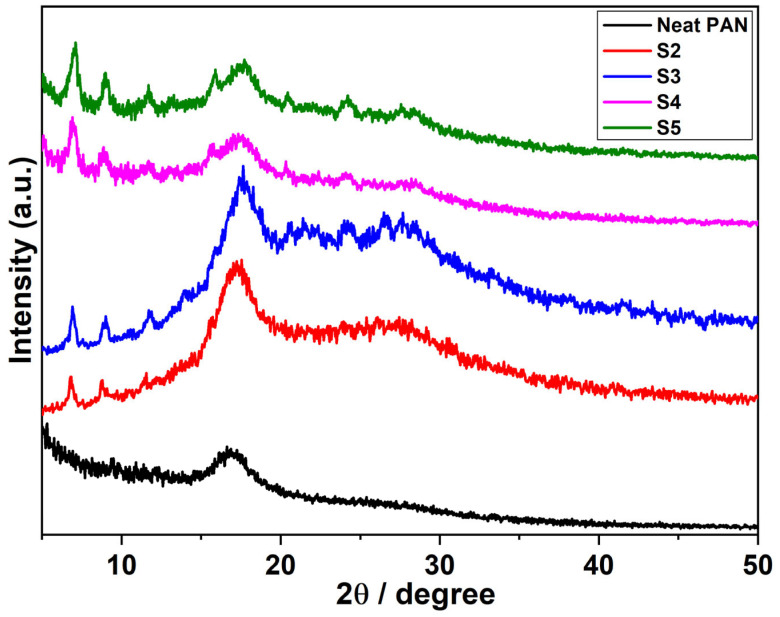
XRD diffractograms of neat (S1) and DLF loaded PAN nanofibers (S2, S3, S4, and S5).
